# Longitudinal retrospective cohort study to examine liver, cardiovascular and metabolic factors as predictors of all-cause mortality in a rural population in South-West Uganda

**DOI:** 10.1136/bmjph-2025-004426

**Published:** 2026-08-03

**Authors:** Cori Campbell, Joseph O Mugisha, Beatrice Kimono, Elizabeth Waddilove, Ronald Makanga, Florence Nambaziira Muzaale, Tingyan Wang, Janet Seeley, Moffat Nyirenda, Philippa C Matthews, Robert Newton

**Affiliations:** 1 NIHR Oxford Biomedical Research Centre, Oxford, UK; 2 Nuffield Department of Medicine, University of Oxford, Oxford, UK; 3 MRC/UVRI and LSHTM Uganda Research Unit, Entebbe, Uganda; 4 The Francis Crick Institute, London, UK; 5 NIHR Oxford Biomedical Research Centre, Big Data Institute, University of Oxford, Oxford, UK; 6 Oxford University Hospitals NHS Foundation Trust, NIHR Health Informatics Collaborative, Oxford, UK; 7 Social Science Unit, Africa Health Research Institute, Durban, South Africa; 8 Department of Global Health & Development, London School of Hygiene and Tropical Medicine, London, UK; 9 NCD Epidemiology, London School of Hygiene & Tropical Medicine, London, UK; 10 Division of Infection and Immunity, University College London, London, UK; 11 Department of Infectious Diseases, University College London, London, UK; 12 Department of Health Sciences, University of York, York, UK

**Keywords:** Hypertension, HIV, Epidemiology, Prevalence, Public Health

## Abstract

**Introduction:**

Markers of liver and metabolic disease have not been well described for many populations in Africa, but could be important to inform individual and public health interventions that reduce morbidity and mortality.

**Methods:**

We studied longitudinal data from a population in rural Uganda, aiming (1) to describe trends in liver function tests and (2) to investigate how liver, metabolic and cardiovascular parameters are associated with all-cause mortality. Demographic and laboratory data were collected through the General Population Cohort in South-Western Uganda. We summarised cohort characteristics at baseline using descriptive statistics and used univariable and multivariable Cox proportional hazards models to investigate factors associated with adjusted HR (aHR) for death over a 12-year period (between 2011 and 2023).

**Results:**

Our dataset includes 7896 individuals, of whom 4441/7896 (56%) were female and 5722/7896 (72%) were aged under 45 years. Prevalence of hepatitis B virus (HBV) was 216/7896 (2.74%; 95% CI 2.38% to 3.10%) and HIV was 582/7896 (7.37%; 95% CI 6.79% to 7.94%). Alanine aminotransferase (ALT) was elevated in 2020/7896 (25.58%; 95% CI 24.62% to 26.55%). During the period observed, all-cause death was associated with older age (p<0.001), male sex (aHR 1.56, 95% CI 1.30 to 1.86) and HIV infection (aHR 1.67, 95% CI 1.25 to 2.22). Mortality was associated with an increase in aspartate aminotransferase:ALT ratio (aHR 1.17, 95% CI 1.08 to 1.26), a marker of alcoholic hepatitis. Increases in systolic blood pressure and haemoglobin A1c were also associated with significantly elevated hazards of death. Under sensitivity analysis restricted to participants with longitudinal follow-up data (n=871), HBV infection was associated with death (aHR 5.38, 95% CI 2.01 to 14.42).

**Conclusions:**

Simple interventions to prevent, diagnose and treat hypertension, diabetes, alcohol excess, and HIV and HBV infection could have an important impact on mortality in this setting.

**WHAT IS ALREADY KNOWN ON THIS TOPIC**

- Liver, metabolic and cardiovascular diseases are an increasing global health priority, but data on epidemiology, aetiology and outcomes are limited for African populations.

**WHAT THIS STUDY ADDS**

- In a rural population in Uganda, increased hazard of death is associated with male sex, hepatitis B virus or HIV infection, increased blood pressure and elevated haemoglobin A1c. We also observe a signal for alcoholic hepatitis linked to mortality.

**HOW THIS STUDY MIGHT AFFECT RESEARCH, PRACTICE OR POLICY**

- This study highlights the need for increased community-based screening and intervention for both communicable and non-communicable diseases to diagnose and tackle common public health threats.

## Introduction

 Health surveillance data are urgently needed to better represent populations in the WHO Africa region in order to inform clinical and public health interventions. In this study, we focus on liver parameters, supplemented by metabolic and cardiovascular measurements and biomarkers, to provide a description of a rural East African population to support a better understanding of community health needs and provide an evidence base for intervention. There is increasing focus on this domain, based on recognition of a growing global burden of liver and metabolic disease which result in high morbidity and mortality.^1^ Globally, the highest age-standardised death rate has been reported in East Africa (at 44.15 per 100 000 population per year).^2^ As screening tools become more widely available and can be offered through decentralised programmes, and opportunities and access to low-cost interventions improve, significant population health benefits can be offered, informed by data representing diverse population settings.

Hepatic enzymes and biomarkers can be measured in serum to assess liver health and have been applied to measuring the global burden of liver disease.^3^ These biomarkers are often collectively referred to as ‘liver function tests’ (LFTs) and include alanine aminotransferase (ALT), aspartate aminotransferase (AST), gamma glutamyl-transferase (GGT), bilirubin (BR) and albumin (Alb). LFT derangement can reflect specific liver pathology, such as viral hepatitis infection, but can also be influenced by systemic perturbations caused by trauma or hypoxia, and be related to extrahepatic or multisystem disorders, including cardiovascular and metabolic disease.^4–8^ Updated terminology defines metabolic dysfunction-associated steatotic liver disease (MASLD) based on hepatic steatosis together with cardiovascular and/or metabolic risk factors^9,10^; a growing prevalence of MASLD is increasingly important as a risk factor for mortality worldwide.^11^

While LFTs may be of utility in characterising liver disease and in predicting outcomes, the extent to which different markers are relevant in different population settings is not clearly established.^12^ Reference ranges for LFTs are not biological absolutes, but have typically been calibrated based on relatively homogenous populations in the global North, based on defining thresholds which capture 95% of a healthy population. However, many such values differ according to ethnicity and can also be influenced by how ‘healthy’ individuals are defined, by the local population environment including diet and endemic disease, and according to assay platforms and calibration.^13–15^ Furthermore, LFT measurement is most commonly undertaken in hospital/secondary care settings, and therefore many studies are not representative of the distribution of LFT values across the whole population.^16^ Hypertension, high low density lipoprotein (LDL)-cholesterol and elevated haemoglobin A1c (HbA1c) are well-recognised risk factors for cardiovascular mortality, and these parameters need scrutiny in rural African populations to improve targeted monitoring and intervention.^17^

We collected and analysed data from a rural East African setting, collected through the Medical Research Council (MRC) General Population Cohort (GPC), based in Kyamulibwa in the South West of Uganda.^18^ Our specific objectives were (1) to describe the baseline and longitudinal distributions of LFTs and (2) to investigate how LFTs, metabolic and cardiovascular parameters in this setting are associated with all-cause mortality. In the longer term, this investigation can be used to help prioritise screening and interventions for conditions with the greatest impact on public health, supporting resource allocation.

## Methods

### Cohort design and participant sample

**Figure 1:**
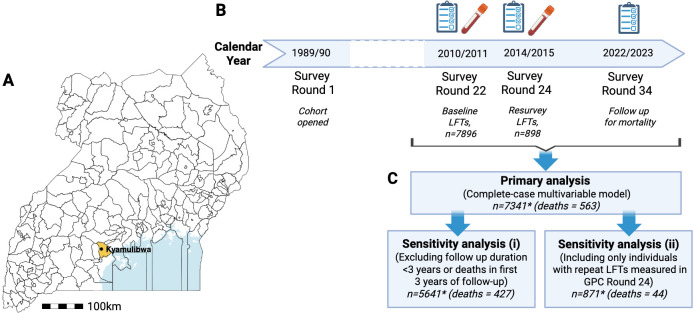
Location and timeline of study of the General Population Cohort (GPC) in Uganda. (A) Map of Uganda, with county lines marked; Kalungu County is highlighted in yellow and the location of the GPC in Kyamulibwa is marked. (B) Time points of data collection for this study are presented, with blood bottle icons indicating time points at which liver function tests (LFTs) were measured (survey rounds 22 and 24). The cohort was established in 1989, with the study population recruited via annual household census rounds. Follow-up for reporting of deaths is available up to the 2022/2023 round for this study (survey round 34). (C) Flow chart to show the number of participants represented in the primary analysis and two sensitivity analyses. * Numbers for each analysis category are lower than the for the total with LFTs tested due to data availability. Map drawn in R, and figure panels B and C generated in BioRender.com with a licence to publish.

The GPC, located in the Kyamulibwa subcounty of the Kalungu district in South West Uganda (figure 1A), is a prospective population cohort under longitudinal follow-up, as previously described.^18–20^ The framework was originally established in 1989 to monitor HIV incidence and prevalence in the region, with an annual census to collect demographic, biometric and clinical data. In most survey rounds, all individuals aged ≥13 years planning to reside ≥3 months in a household located within the study region have been eligible for inclusion, with age restrictions relaxed to include younger participants in certain survey rounds. GPC participants have been prospectively followed up to detect relevant endpoints, and participant data from the survey are linked to data collected at the Medical Research Council (MRC)/Uganda Virus Research Institute (UVRI) clinic via unique individual identifiers. The clinic provides care to participants with acute medical conditions, along with some chronic morbidities including HIV infection and diabetes mellitus. Survey rounds have been consecutively numbered since the start of the cohort, and in this study we draw on data from survey rounds 22 (years 2011–2012) and round 24 (years 2014–2015), which we defined as ‘baseline’ and ‘re-survey’ time points, respectively (figure 1B). Children aged 3–12 years were included in round 22, however, round 24 was restricted to those aged ≥13 years.

Each participant was assigned a unique GPC identifier at initial enrolment, which was used consistently across all subsequent medical survey rounds and each annual census.

### Covariate data collection and thresholds

Data collected in each survey round include demographic factors, biophysical measurements (height, weight, blood pressure and body mass index (BMI, kg/m^2^)).

#### Sociodemographic and biometric data

Sociodemographic data were gathered by interviewers who administered questionnaires to a household head or any adult representative. Age is presented as categorical data to allow the presentation of summary statistics by age class and to allow for potential non-linear associations with the outcome and to improve interpretability of the model estimates.

The WHO STEPwise Approach was used to collect cardiovascular risk data (applying step-by-step instructions in the modules for physical measurements).^21^ Biophysical measurements (blood pressure, weight, height, waist and hip circumferences) were performed using standardised procedures as previously described^22,23^; BMI between 18.5 and 24.9 kg/m^2^ was classified as healthy weight, with <18.5 being underweight, and >24.9 overweight. Further details regarding measurement of these parameters in supplemental file S1.

#### Laboratory data

LFTs (serum AST, ALT, ALP, GGT and BR) were measured in a clinically validated diagnostic laboratory in Entebbe, using a Cobas Integra 400 plus machine, with reagents supplied by Roche and protocols according to the manufacturer’s instructions. Methods for testing for Blood Borne Virus Infection (HIV, hepatitis B virus (HBV) and HCV) are reported in online supplemental file S2. For this study, we used LFT data from survey round 22 and round 24 (figure 1B). We also included round 22 data for metabolic biomarkers (including HbA1c, LDL, high density lipoprotein (HDL) and triglycerides) and screening for HIV and HBV infection. Laboratory analysis of blood samples was undertaken at intermittent time points, subject to availability of funding (not all parameters were measured at every survey round due to cost implications).

We used two definitions of LFT derangement to investigate associations with all-cause mortality, (1) applying published ALT thresholds,^24^ defined as serum concentrations above the upper limit of normal (ULN) as >19 U/L in females and >30 U/L in males (these thresholds have also been employed by international WHO HBV guidelines^25^) and (2) calculating AST:ALT ratio; where a ratio >2 is an accepted marker of possible alcoholic hepatitis.^26,27^ We excluded individuals ≤19 years from ALP analysis, since elevated ALP can be attributable to bone growth in teenagers.

### Follow-up and ascertainment of death

The primary outcome for this analysis was death from any cause, which was ascertained from mortality registers, verbal autopsy reports and reports from community health workers as previously described in other publications.^20,28,29^ Monthly mortality registers were populated by village recorders resident in the GPC study villages. These data were supplemented with deaths identified through the annual census to ensure complete ascertainment of mortality events. For participants who died, the GPC identifier was also used to link records from the mortality registers and verbal autopsy data with medical survey data, enabling reliable linkage across data sources. Mortality registers were updated on a monthly basis over the time period studied up to survey round 34.

### Statistical analysis

We summarised cohort characteristics at baseline using descriptive statistics, summarising continuous laboratory parameters with means and SD, or medians and IQRs and by division using quintiles. We used univariable and multivariable Cox proportional hazards models to investigate factors associated with hazards of all-cause mortality.

Our primary multivariable model used baseline data (GPC round 22) from participants who had complete information regarding demographic factors, biophysical measurements and markers quantified from blood samples (including LFTs and metabolic biomarkers). Factors were included in multivariable models according to both significance of univariable associations (where p≤0.1) and/or based on biological/clinical relevance and previous literature from both the GPC^18,28^ and investigations undertaken in differing patient populations.^5,6,8,30–32^ This p-value threshold of 0.1 strikes a balance: we aim to avoid prematurely excluding potentially important variables (because univariate analyses may have limited power to detect modest but meaningful associations, particularly in the presence of confounding, a relaxed threshold allows variables with possible clinical or biological relevance to be retained for evaluation in the multivariable model). A higher p-value threshold would be more likely to lead to spurious associations and added noise. We calculated univariable HRs and multivariable adjusted HR (aHR), along with 95% CI, as model outputs. Statistical analyses were performed in R (V.4.1.0).

To interrogate the association of BMI with HbA1c in this population, the association between the two variables was visualised, and a nonlinear relationship was fit using a generalised additive model (online supplemental file S5).

### Sensitivity analyses

We performed two sensitivity analyses. First, the multivariable Cox proportional hazards model excluded patients who died within 3 years of baseline LFT measurement, to account for the influence of reverse causality bias on the findings from the main model, also excluding those with <3 years follow-up. Second, we analysed the subset of patients who had LFTs measured at both our baseline assessment (GPC round 22; years 2010–2011) and resurvey time point (GPC round 24; years 2014–2015), to compare the robustness of main model results against a model accounting for longitudinal changes in LFTs. For this, we used a time-updated Cox proportional hazards model to incorporate changes in LFT serum concentrations over time, whereby follow-up time was split according to measured LFT concentrations and updated when a change in concentration was reported. Datasets used in the primary analysis and sensitivity analyses are shown in figure 1C.

### Reporting guidelines

To ensure consistency of reporting, we have included a STROBE (STrengthening the Reporting of OBservational studies in Epidemiology) statement (online supplemental file S3).

### Patient and public involvement

The research has been supported by an active community engagement programme led from the Kyambulibwa site from the outset of the GPC. Clinical and academic team members work with members of the community to provide health advice and information about research projects and to address questions and collate feedback to ensure representation of the community and to align the programme with local population needs and priorities. This includes dialogue events in which members of the community gather with an open invitation to attend. All information is presented in the local language (Luganda). Specific examples of this activity are available in a project report which describes ongoing prospective work on liver disease in this setting.^33^

### Data availability

A data dictionary and data availability can be accessed through the London School of Hygiene and Tropical Medicine Data Compass (https://datacompass.lshtm.ac.uk/id/eprint/4497/; DOI: 10.17037/DATA.00004497).^34^ All data (census, survey, mortality and laboratory) generated through the cohort are stored and curated at the MRC/UVRI and the LSHTM Research Unit. Based on governance frameworks for GPC data protection, there are restrictions on access to the underlying data. However, data access for specific research purposes is possible; further information on data access can be found online here: https://apps.mrcuganda.org/mrcdatavisibility/home/gpc/.

## Results

### Baseline characteristics

At baseline, we included data representing 7896 individuals (table 1). There was a slight female excess (4441/7896, 56.2%) and participants aged <45 years accounted for the majority (5722/7896, 72.5%). BMI ranged between 18.5 and 24.9 kg/m^2^ for the majority (4769/7896, 64.8%), representing a healthy range. HBV infection was present in 216/7896 (2.74%; 95% CI 2.38% to 3.10%) and HIV in 582/7896 (7.37%; 95% CI 6.79% to 7.94%). Participants were followed up for a mean 6.4 (SD 3.2) years. At baseline, ALT elevation >ULN was present in 2020/7896 (25.6%; 95% CI 24.62% to 26.55%) and AST:ALT ratio was >2 in 857/7896 (10.9%; 95% CI 10.17% to 11.54%).

**Table 1:** Baseline characteristics for a subset of individuals included in the Uganda MRC general population for whom liver function test measurement was performed in survey round 22

Characteristic	Total	Died	Survived
Number of participants	7896	629 (7.97)	7267 (92.03)
Sex			
Male, n (%)	3455 (43.8)	340 (54.1)	3115 (42.9)
Age group, n (%)			
Age (years), mean (SD)	34.01 (18.36)	59.4 (19.5)	31.8 (16.5)
<18 years	1982 (25.1)	26 (4.1)	1956 (26.9)
18–24 years	1198 (15.2)	15 (2.4)	1183 (16.3)
25–34 years	1379 (17.5)	48 (7.6)	1331 (18.3)
35–44 years	1163 (14.7)	53 (8.4)	1110 (15.3)
45–54 years	936 (11.9)	75 (11.9)	861 (11.8)
55–64 years	578 (7.3)	107 (17.0)	471 (6.5)
65–74 years	414 (5.2)	152 (24.2)	262 (3.6)
≥75 years	246 (3.1)	153 (24.3)	93 (1.3)
Liver function tests			
ALT>ULN, n (%)	2020 (25.6)	140 (22.3)	1880 (25.9)
AST:ALT ratio, median (IQR)	1.43 (1.19, 1.71)	1.64 (1.34, 1.96)	1.42 (1.18, 1.68)
AST:ALT ratio >2, n (%)	857 (10.9)	147 (23.4)	710 (9.8)
ALP, median (IQR)	93 (71, 144)	91 (75, 116)	93 (71, 151)
AST>ULN, n (%)	635 (8.0)	92 (14.6)	543 (7.5)
Albumin, median (IQR)	41.70 (39.25, 43.85)	38.89 (35.00, 41.69)	41.89 (39.57, 44.00)
Lipid profile			
Cholesterol, median (IQR)	3.41 (2.84, 4.07)	3.63 (2.94, 4.36)	3.39 (2.84, 4.05)
HDL, median (IQR)	0.96 (0.74, 1.20)	1.02 (0.78, 1.32)	0.95 (0.73, 1.19)
LDL, median (IQR)	1.93 (1.50, 2.43)	2.00 (1.44, 2.58)	1.92 (1.50, 2.43)
Triglycerides, median (IQR)	1.05 (0.78, 1.43)	1.14 (0.85, 1.55)	1.04 (0.78, 1.41)
HbA1c, median (IQR)	3.33 (2.95, 3.70)	3.45 (3.04, 3.85)	3.32 (2.94, 3.69)
BMI			
BMI (kg/m^2^), mean (SD)	20.66 (18.71, 22.90)	19.74 (17.78, 22.22)	20.70 (18.79, 22.95)
<18.5 kg/m^2^, n (%)	1678 (22.8)	180 (31.9)	1498 (22.0)
18.5–24.9 kg/m^2^, n (%)	4769 (64.8)	324 (57.3)	4445 (65.4)
25–29.9 kg/m^2^, n (%)	718 (9.8)	42 (7.4)	676 (9.9)
30–34.9 kg/m^2^, n (%)	147 (2.0)	13 (2.3)	134 (2.0)
≥35 kg/m^2^, n (%)	50 (0.7)	6 (1.1)	44 (0.6)
BP (mm Hg)			
Average systolic BP, median (IQR)	120 (112, 130)	131 (118, 152)	119 (111, 129)
Average diastolic BP, median (IQR)	73 (68, 80)	78 (71, 87)	73 (67, 80)
HIV status			
Negative, n (%)	7314 (92.6)	564 (89.7)	6750 (92.9)
Positive, n (%)	582 (7.4)	65 (10.3)	517 (7.1)
HBV status			
Negative, n (%)	7680 (97.3)	611 (97.1)	7069 (97.3)
Positive, n (%)	216 (2.7)	18 (2.9)	198 (2.7)
Longitudinal follow-up available with LFTs repeated in GPC round 24			
Resurveyed, n (%)	898 (11.4%)	45 (7.2%)	853 (11.7%)

Characteristics are reported overall and by survival status after a mean follow-up of 6.4 years undertaken between 2010 and 2023.

ALP, alkaline phosphatase; ALT, alanine aminotransferase; AST, aminotransferase; BMI, body mass index; BP, blood pressure; GPC, General Population Cohort; HbA1c, haemoglobin A1c; HBV, hepatitis B virus; HDL, high density lipoprotein; LDL, low density lipoprotein; LFT, liver function test; ULN, upper limit of normal based on published thresholds.

### Longitudinal LFT trends

LFTs were quantified at re-survey in 898/7896 (11.4%) participants (table 2; figure 1B). Median values of ALT, AST and GGT had fallen compared with baseline, and thus smaller proportions of participants had ALT>ULN at follow-up (28.7% vs 13.8%, p<0.001). In keeping with the decline in ALT, the median AST:ALT ratio was higher at resurvey, with an increase in the proportion of the population with ratio >2, from 11.2% at baseline to 36.0% at resurvey (p<0.001).

**Table 2:** Liver function tests (LFT) in the Uganda General Population Cohort, divided according to those under longitudinal follow-up and those with baseline measurement only

Characteristic	Measurement at baseline	Measurement at follow-up
Abnormal ALT, n (%)		
Participants with follow-up LFT data	258 (28.7)	122 (13.6)***
Participants with baseline only LFT data	1762 (25.2)	N/A
ALT, median (IQR)		
Participants with follow-up LFT data	18.23 (14.19,23.55)	11.30 (6.70, 17.28)
Participants with baseline only LFT data	17.72 (13.96, 22.78)	N/A
AST, median (IQR)		
Participants with follow-up LFT data	25.64 (21.76, 31.22)	19.50 (13.70, 25.90)
Participants with baseline only LFT data	25.08 (21.13, 30.27)	N/A
AST:ALT ratio, median (IQR)		
Participants with follow-up LFT data	1.44 (1.20, 1.71)	1.73 (1.34, 2.30)
Participants with baseline only LFT data	1.42 (1.17, 1.72)	N/A
AST:ALT ratio >2, n (%)		
Participants with follow-up LFT data	101 (11.2)	323 (36.0)***
Participants with baseline only LFT data	756 (10.8)	N/A
GGT, median (IQR)		
Participants with follow-up LFT data	19.22 (14.05, 28.20)	15.00 (10.00, 23.00)
Participants with baseline only LFT data	18.72 (13.46, 27.80)	N/A

Baseline data were collected for n=7896, divided here into those with follow-up measurements (n=898) and baseline survey data only (n=6998).

*p<0.05; **p<0.01; ***p<0.001; P values are for the difference in proportion between those measured at baseline and measured at follow-up.

ALT, alanine aminotransferase; AST, aspartate aminotransferase; GGT, gamma glutamyl-transferase; N/A, not applicable.

### Risk factors for all-cause mortality

**Figure 2:**
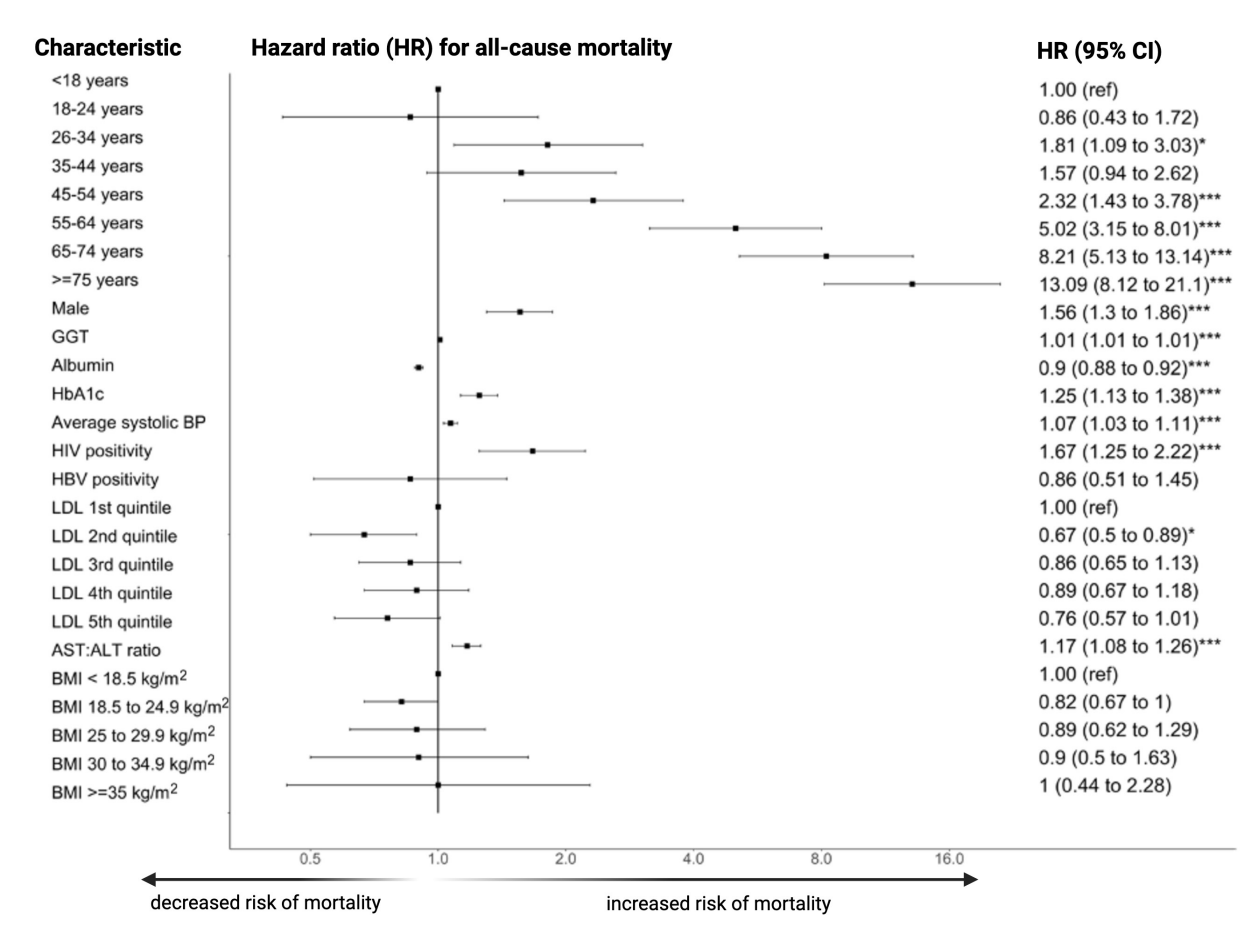
Forest plot for Cox proportional hazards model to identify risk factors for all-cause mortality in the Uganda General Population Cohort study. The plot represents data for 7341 individuals, over a follow-up period of a mean of 6.4 years (SD 3.2 years), among whom there were 563 deaths. This plot includes the estimates for the most parsimonious multivariable model (parameters excluded if univariable associations not significant). ALT, alanine aminotransferase; AST, aspartate aminotransferase; BMI, body mass index; BP, blood pressure; GGT, gamma-glutamyl transferase; HbA1c, glycated haemoglobin; HBV, hepatitis B virus; LDL, low-density lipoprotein; ref, reference. * *P* < 0.05; ** *P* < 0.01; *** *P* < 0.001

The main complete-case multivariable model (using baseline data) included 7341 participants, of whom 563/7341 (7.7%) died throughout follow-up. We identified risk factors for all-cause mortality (figure 2). As expected, mean age was older (59.4 years, SD 19.5) in those who died as compared with those who survived (31.8 years, SD 16.5) (p<0.001). Increased hazards of death during the period of observation were also associated with male sex (aHR 1.56, 95% CI 1.3 to 1.86) and HIV infection (aHR 1.67, 95% CI 1.25 to 2.22).

A one unit increase in AST:ALT ratio was associated with 32% increase in death hazards (aHR 1.17, 95% CI 1.08 to 1.26), while 10-unit increases in serum GGT were associated with 1% increased hazards (aHR 1.01, 95% CI 1.01 to 1.01). Both of these markers are associated with alcohol excess. Conversely, hazards were reduced with increased serum albumin, for which a one unit increase predicted a 10% reduction in hazards of death (aHR 0.86, 95% CI 0.85 to 0.87).

On assessment of cardiovascular and metabolic factors, a 10 mm Hg increase in systolic blood pressure was associated with 7% increased hazards of death (aHR 1.07, 95% CI 1.03 to 1.11), and one unit increases in serum HbA1c were associated with 25% increased hazards of death (aHR 1.25, 95% CI 1.13 to 1.38). Although HbA1c is associated with BMI (online supplemental file S4), there was no statistically significant relationship between BMI and death in this population. Reduced hazards of death were observed in the second LDL quintile (aHR 0.67, 95% CI 0.5 to 0.89) compared with the first quintile, but there were no associations for the other quintile groups.

### Sensitivity analyses

The first sensitivity analysis undertaken was based on data which excluded deaths occurring within the first 3 years of follow-up (n=5641) (online supplemental file S5). In general, the associations observed in the main multivariable model remained unchanged in terms of both direction and magnitude. However, the association of HIV positivity with increasing hazards of death was attenuated towards the null.

The second sensitivity analysis undertaken was restricted to the smaller set of participants with longitudinal LFT measurement (n=871), and utilised a time-updated Cox proportional hazards model to incorporate changes in LFT serum concentrations over time (online supplemental file S5). Most associations observed in the main multivariable model remained unchanged in direction. CIs for all associations were widened due to the smaller number of observations. AST:ALT ratio and HbA1c were no longer significantly associated with mortality in this sensitivity analysis. However, HBV-positive status became significant under sensitivity analysis, with a HR for mortality of 5.38 (95% CI 2.01 to 14.42) (online supplemental file S5).

## Discussion

### Clinical and public health relevance and rationale

In this young rural Ugandan population, we had the unique opportunity to undertake analysis of longitudinal follow-up in a large cohort, underlining co-endemic HIV and HBV infection, alcohol and cardiovascular factors as important and modifiable risk factors for mortality. Liver biomarkers that can help to stratify population health include AST:ALT ratio, GGT and albumin. Hypertension represents a public health concern here, as in many African populations, but noting that region-specific differences are influenced by population genetics, urban/rural location, diet and comorbidity.^35,36^ The excess risk of male sex suggests the need for ongoing efforts to target education and interventions towards boys and men, who typically have fewer points of contact with healthcare services than their female counterparts.

Liver, metabolic and cardiovascular-related morbidity and mortality are poorly studied in African populations, despite representing a growing public health problem at the intersection of infection and non-communicable disease. In a worldwide study of >1.5 million individuals, five modifiable risk factors have been reported to account for ~20% of deaths (BMI, systolic BP, non-HDL cholesterol, smoking and diabetes).^37^ As all of these parameters are also potentially associated with liver disease, and there is growing concern about the global burden of MASLD, we highlight the imperative to collect data representing diverse populations to inform public health strategy. Markers that are associated with mortality and can be measured simply in community settings may help to inform targeted health interventions for education, screening, lifestyle interventions and treatment.

African populations are highly diverse in terms of genetics, risk of infection and other comorbidity, diet and alcohol intake, environmental exposures and access to healthcare. Many of these influences also change over time, and in association with climate change, migration and urbanisation. While our analysis identifies trends which may be important across many settings, generalisation should be undertaken cautiously to account for diverse influences on liver and metabolic health and mortality.

### Impact of alcohol, metabolic disease and serum albumin on liver health

The combination of alcoholic liver disease (ALD), with metabolic or steatotic liver pathology (‘MetALD’) is increasingly recognised as a global public health concern.^38^ The increase in the proportion of our Ugandan cohort with AST:ALT>2 over time, together with a modest but statistically significant relationship between GGT elevation and mortality, is congruent with our previous findings suggesting a high prevalence of alcohol-related liver disease and liver fibrosis in the GPC population.^39^ The prevalence and outcomes of ALD and MetALD need further investigation in this setting.

We report a reduction in hazards of death associated with increasing serum albumin concentrations. This finding is expected on biological grounds, as low albumin is associated with ill health (eg, renal or gut disease, cancer, chronic infection or inflammation) and therefore more likely to be associated with higher mortality; thus, an increase in albumin is protective. This observation has also been reported in a UK dataset.^6^

### Chronic viral infection, liver disease and mortality

Despite substantial advances in HIV diagnosis, treatment and care, HIV infection in this population was still associated with a significant increase in mortality during the time-course of this study. However, it should be noted that further improvements have continued over time, and updated investigations will be required to determine whether this association has diminished as access to diagnosis and treatment has been strengthened.

HBV has an effect on mortality due mainly to cirrhosis and hepatocellular carcinoma, and is a neglected public health threat in many African populations.^40,41^ A significant association between HBV and mortality emerged in our sensitivity analysis (HR 5.38), and our team has also followed up people living with HBV in this population to better understand the characteristics of chronic infection.^42^ New WHO guidelines support expanded diagnosis and relaxed treatment eligibility criteria for people living with HBV,^25^ and this signal for the relationship with mortality in rural Uganda highlights the pressing case of need for scale-up of HBV testing and treatment. Our data represent an important opportunity for high-profile advocacy for maintaining access to diagnosis, monitoring and treatment in African populations, despite the withdrawal of programmes such as PEPFAR (the United States President’s Emergency Plan for AIDS Relief) and USAID (the United States Agency for International Development) which have provided life-saving infrastructure, consumables and medications over the past two decades.^43^

### Caveats and limitations

Due to the retrospective nature of the study, cost limitations on what data could be collected in each survey round, and lack of access to many diagnostics in this rural resource-limited setting, the study does not capture all major causes of mortality. Although the GPC aims to represent a cross-section of the whole population, we recognise sources of bias and missingness, as people who are unwell may be less willing or able to enrol in the cohort, and there is migration in and out of the area. At follow-up, LFTs were only measured in a subset of the cohort, due to the cost of running these tests, which affects the power of the study and may account for differences in results between the main and sensitivity analyses. In Uganda, as in much of the WHO African region, and particularly for rural regions, there is no gold standard approach to reporting mortality and our current approach is the most rigorous possible method to capture all deaths over the time period studied, recognising that some deaths could have been missed. We report mortality as a binary outcome, rather than interrogating local records or verbal autopsies for cause of death.

Thresholds for laboratory blood markers and biophysical measurements have not been thoroughly validated in African populations; commonly used thresholds for LFT derangement have been developed in discrete and homogenous populations (more typically in the global North and in high income settings),^24,44^ and therefore may not be sensitive or specific when applied elsewhere.^39^

Our analysis is based on historic data collection, and there is a need for review of new data in order to determine which of the effects we observe remain true over time. We have described associations for which we can offer potential explanations, but factors underlying these associations cannot be definitively determined through this study, and there may be unmeasured confounding that influences our results. We used all-cause mortality as an endpoint, but further granularity could be added to analysis if causes of death were available; this information was not accessible for this study.

All liver biomarkers are non-specific. Although elevated GGT is often considered a marker of alcohol excess, GGT levels have also been associated with abnormal cardiometabolic variables in a South African study of people living with HIV,^45^ and a change in AST:ALT ratio over time can be driven by improvements in ALT as well as worsening AST. It is therefore important to consider changes in these biomarkers together with other changes in population health over time. Likewise, albumin is a non-specific marker, which when low can be a marker of inadequate nutrition,^46^ but is also associated with uncontrolled chronic infection, trauma, malignant or inflammatory disease,^47^ and may reflect gut, liver and/or renal disease. Due to the multiple influences that determine serum albumin concentration, there is no single intervention that could be used to target hypoalbuminaemia unless its root causes are more specifically explored.

In this analysis, although HbA1c is associated with increased mortality, population values were low overall. We were underpowered to assess the impact of overweight/obesity due to its low prevalence in the population studied, which may account for the absence of a statistically significant relationship between BMI and mortality in this dataset.

Collection of data on alcohol consumption is important but challenging, as self-reporting is known to be inaccurate, and locally brewed drinks contain varying alcohol concentrations. Locally available alcoholic beverages may also contain other hepatotoxins^48^ which contribute to the acceleration of liver disease.

Assessment of hepatic steatosis requires either a liver biopsy (which is invasive, expensive and carries some clinical risk) or can be quantified using a range of non-invasive tests, including Controlled Attenuation Parameter measurement, for example, using FibroScan technology (EchoSens, Paris). The latter is appealing as it is a quick and painless assessment, for which limited operator training is needed (and subsequent to the timeline of this study) we have introduced this tool for measurement of liver stiffness and steatosis in Uganda^49^ as well as in Kenya and South Africa.^50^ However, access to Fibroscan for many settings is currently constrained by the high costs of acquiring and maintaining the hardware.

The associations of increasing age and HIV with increasing hazards of death were attenuated towards the null in the first sensitivity analysis, especially for the older age categories. This likely reflects the influence of reverse causality bias as older participants and those with HIV infection might have had clinically symptomatic and/or advanced disease at recruitment. Only a minority of participants with longitudinal LFT measurement were available for inclusion in sensitivity analyses, which affected our statistical power to detect associations.

## Conclusions

In this rural African population, risk factors for all-cause mortality include age, male sex, hypertension, alcohol excess, HIV and HBV infection. Investing in enhanced assessment of liver health, including viral hepatitis, MASLD, ALD and MetALD is important, as all of these conditions can be tackled through education and public health interventions, with many opportunities for prevention as well as treatment. Male vulnerability may need to be tackled by encouraging and supporting men to attend for screening and health interventions. Further work is needed to determine the extent to which these parameters have continued to influence mortality, in order to inform the evidence-based delivery of community health programmes.

## Supplementary material

10.1136/bmjph-2025-004426 supplemental file S1-S5

## Data Availability

Data availability is subject to governance terms of the Uganda GP/ London School of Hygiene and Tropical Medicine. Further information can be found online here: https://apps.mrcuganda.org/mrcdatavisibility/home/gpc/.
