# Supplementary material for: Longitudinal retrospective cohort study to examine liver, cardiovascular and metabolic factors as predictors of all-cause mortality in a rural population in South-West Uganda
Source: BMJ Public Health. 2026 Aug 3;4(3):e004426. doi: 10.1136/bmjph-2025-004426 (PMC13436094; doi:10.1136/bmjph-2025-004426)
Supplement: Supplementary file 1 [file bmjph-4-3-s001.pdf]

SUPPLEMENTARY MATERIAL (S1-S5)

A longitudinal retrospective cohort study to examine liver, cardiovascular and metabolic factors as predictors of all-cause mortality in a rural population in South-West Uganda

CONTENTS OF SUPPLEMENT

S1: Standardised approach to blood pressure and BMI measurement 2

S2: Standardised approach to testing for blood borne virus infection 3

S3. STROBE Statement [Strengthening the reporting of observational studies in epidemiology] for Liver, cardiovascular and metabolic factors as predictors of all-cause mortality in a rural Ugandan Cohort... 4

S4. Relationship between body mass index and HbA1c in the Uganda GPC. 8

S5. Cox proportional hazards model for mortality based on univariable associations, complete case main model (n = 7341, deaths = 563) and sensitivity analyses.................... 9

S1: Standardised approach to blood pressure and BMI measurement

In the WHO STEPwise Approach to Surveillance (STEPS) protocol, blood pressure and body mass index (BMI) are core physical measurements collected in **Step 2** (Physical Measurements).

Full details are available here:
https://www.who.int/docs/default-source/ncds/ncd-surveillance/steps/steps-manual.pdf

In brief, blood pressure was measured on the right arm using appropriate cuff sizes (regular arm cuff size if arm circumference was 24-32cm, large arm cuff if arm circumference was 33-41cm, thigh cuff if over 41cm and if under 24cm paediatric cuff size was used) in a sitting position, three times within resting intervals of 5 minutes, using a digital sphygmomanometer (the Omron M4-I). The mean of the second and third reading was taken for analysis. Fixed hypertension thresholds are defined (≥140/90 mmHg in classic STEPS reporting).

Anthropometry
Body weight was measured using the Seca 761 mechanical scales and body height was measured using a portable Leicester stadiometer to the nearest 1 kg and 0.1 cm respectively, with participants wearing light clothing and no shoes.

BMI is categorised as follows (WHO adult cut-offs):

- <18.5 — Underweight
- 18.5–24.9 — Normal
- 25.0–29.9 — Overweight
- ≥30 — Obese

All Step 2 measurements (BP, height, weight, waist) are designed and validated for adults. Standard STEPS surveys typically include ages 18–69, however in this case was also used in older adults. Children require age- and sex-specific BMI z-scores or percentiles, and WHO child references are applied instead.

S2: Standardised approach to testing for blood borne virus infection

HIV testing was done using an algorithm recommended by the Uganda Ministry of Health, starting with screening with a locally approved rapid diagnostic test (RDT). If the test result was negative, the participant was considered to be HIV negative. If the test result was positive, the sample was retested with the rapid test HIV-1 or HIV-2 Stat-Pak. If both tests resulted in a positive result, the participant was diagnosed as living with HIV infection. If the tests gave discordant results, the sample was further evaluated with the rapid test Uni-Gold Recombinant HIV-1/2. For those samples assessed by all three tests, two positive test results were interpreted as positive, and two negative results were considered negative.

HBV surface antigen (HBsAg) testing was conducted using Cobas HBsAg II (2011–08 V.10), and those who tested positive were invited for further serologic testing.

HCV was tested using a combination of immunoassays followed by PCR, as previously described*.

* O'Hara G, et al. Liver function tests and fibrosis scores in a rural population in Africa: a cross-sectional study to estimate the burden of disease and associated risk factors. BMJ Open. 2020 ;10(3):e032890. Available from: https://bmjopen.bmj.com/content/10/3/e032890

S3. STROBE Statement [Strengthening the reporting of observational studies in epidemiology] for Liver, cardiovascular and metabolic factors as predictors of all-cause mortality in a rural Ugandan Cohort

Section in Paper	Item No.	Recommendation	Notes
**Title and abstract**	1	(*a*) Indicate the study's design with a commonly used term in the title or the abstract.	Longitudinal retrospective cohort study indicated in title.
		(*b*) Provide in the abstract an informative and balanced summary of what was done and what was found.	Structured abstract presented to include background, methods, key findings and rationale. Sensitivity analysis included.
Introduction			
**Background/ rationale**	2	Explain the scientific background and rationale for the investigation being reported.	Introduction explains case of need for data representing liver and cardiometabolic health in African populations, with references underpinning the rationale.
**Objectives**	3	State specific objectives, including any prespecified hypotheses.	Specific numbered aims are stated in the final paragraph of the introduction section.
Methods			
**Study design**	4	Present key elements of study design early in the paper.	Study design presented in methods, including setting the scene with cohort design and participant sample.
**Setting**	5	Describe the setting, locations, and relevant dates, including periods of recruitment, exposure, follow-up, and data collection.	Details included in methods. Figure 1 shows the location (panel A), timeline (panel B) and subgroup analyses (panel C).
**Participants**	6	(*a*) Give the eligibility criteria, and the sources and methods of selection of participants. Describe methods of follow-up.	Details included in methods and through reference to other papers which describe analyses undertaken within the same population cohort. Follow-up dates are presented in Figure 1B.
		(*b*) For matched studies, give matching criteria and number of exposed and unexposed.	N/A

**Variables**	7	Clearly define all outcomes, exposures, predictors, potential confounders, and effect modifiers. Give diagnostic criteria, if applicable	Relevant markers described in methods section. Ascertainment of primary outcome (mortality) described in methods.
**Data sources / measurement**	8	For each variable of interest, give sources of data and details of methods of assessment (measurement). Describe comparability of assessment methods if there is more than one group	Measurements, variables and reference ranges (where relevant) stated in methods. Further details of biomarkers, measurements and thresholds are available in the supplement (section S1).
**Bias**	9	Describe any efforts to address potential sources of bias.	Sensitivity analysis tackles potential reverse causality bias. Bias and missingness are addressed in the discussion (subsection which tackles caveats and limitations).
**Study size**	10	Explain how the study size was arrived at.	N/A – we used full data from a community population cohort (described in methods).
**Quantitative variables**	11	Explain how quantitative variables were handled in the analyses. If applicable, describe which groupings were chosen and why.	Handling of quantitative data is described in methods and supplement (S1). This includes use of standardised thresholds, with supporting references.
**Statistical methods**	12	(*a*) Describe all statistical methods, including those used to control for confounding.	Statistical analysis is a specific section of the paper's methods.
		(*b*) Describe any methods used to examine subgroups and interactions.	Presentation of data for subgroups is set out in the methods and in Figure 1C. Sensitivity analysis was undertaken to test robustness of the main analysis to reverse causality bias.
		(*c*) Explain how missing data were addressed.	Statistical approaches presented in methods. Sensitivity analysis undertaken in participants who were resurveyed after baseline measurement.
		(*d*) If applicable, explain how loss to follow-up was addressed.	Follow-up is presented in Figure 1B, with explanation in methods. Discussion addresses sources of missingness based on individuals who do not participate.

		(*e*) Describe any sensitivity analyses.	Sensitivity analyses described in methods, Fig 1C, and results.
Results			
**Participants**	13	(a) Report numbers of individuals at each stage of study; e.g. numbers potentially eligible, examined for eligibility, confirmed eligible, included in the study, completing follow-up, and analysed.	Numbers are described and presented in results tables and Figure 1C.
		(b) Give reasons for non-participation at each stage.	Reasons for non-participation are addressed in the discussion.
		(c) Consider use of a flow diagram.	Figure 1B presents a Flow diagram (timeline) and Figure 1C presents a flow of individuals through the study.
**Descriptive data**	14	(a) Give characteristics of study participants (e.g. demographic, clinical, social) and information on exposures and potential confounders.	Descriptive data are presented in Table 1, with key variables listed in results text.
		(b) Indicate number of participants with missing data for each variable of interest.	Number of participants under follow up presented in Table 2, and in Figure 1C
		(c) Summarise follow-up time (eg, average and total amount).	Follow-up time presented in results and in Figure 1B and C.
**Outcome data**	15	Report numbers of outcome events or summary measures over time.	Outcome events (mortality) presented in results.
**Main results**	16	(*a*) Give unadjusted estimates and, if applicable, confounder-adjusted estimates and their precision (eg, 95% confidence interval). Make clear which confounders were adjusted for and why they were included.	Data are presented in tables and in Figure 2 with confidence intervals.
		(*b*) Report category boundaries when continuous variables were categorized.	Presented in methods section and in supplementary data (S1).
		(*c*) If relevant, consider translating estimates of relative risk into absolute risk for a meaningful time period.	N/A

**Other analyses**	17	Report other analyses done—e.g. analyses of subgroups and interactions, and sensitivity analyses.	Sensitivity analyses conducted and presented in supplementary table S5.
Discussion			
**Key results**	18	Summarise key results with reference to study objectives.	Presented in results sections and reinforced in discussion.
**Limitations**	19	Discuss limitations of the study, taking into account sources of potential bias or imprecision. Discuss both direction and magnitude of any potential bias	Limitations, caveats and potential bias are presented in discussion section.
**Interpretation**	20	Give a cautious overall interpretation of results considering objectives, limitations, multiplicity of analyses, results from similar studies, and other relevant evidence.	Presented in discussion, including references to other comparable studies and also addressing the potential benefits and also caveats of generalisation to other settings.
**Generalis-ability**	21	Discuss the generalisability (external validity) of the study results.	Presented in discussion section.
Other information			
**Funding**	22	Give the source of funding and the role of the funders for the present study and, if applicable, for the original study on which the present article is based.	Funding information is presented at the end of the article, including grant reference numbers where applicable.

S4. Relationship between body mass index and HbA1c in the Uganda GPC.

As anticipated, as BMI increases, so does HbA1c, but HbA1c values in this population are low (confidence intervals do not span the clinically important thresholds of 5.7% which represents a cut-off for pre-diabetes and 6.5% which indicates diabetes).

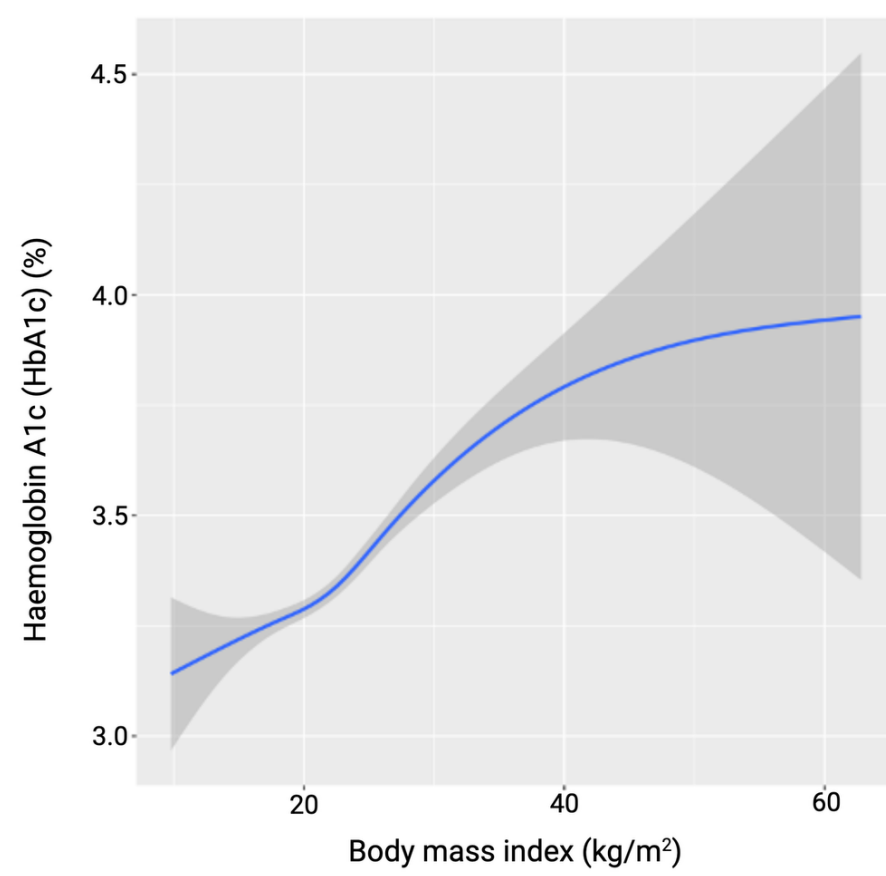

S5. Cox proportional hazards model for mortality based on univariable associations, complete case main model (n = 7341, deaths = 563) and sensitivity analyses.

Sensitivity analysis I: excluding deaths occurring in the first 3 years of follow-up (n = 5641, deaths = 427).
Sensitivity analysis II: restricted to participants with longitudinal LFT measurement (n = 871, deaths = 44).

Characteristic	Univariable HR (95% CI)	Multivariable HR (95% CI)	Multivariable HR sensitivity analysis I, (95% CI)	Multivariable HR sensitivity analysis II, (95% CI)
Age group, n (%)				
<18 years	1.00 (ref)	1.00 (ref)	1.00 (ref)	1.00 (ref)
18-24 years	0.89 (0.47 to 1.69)	0.86 (0.43 to 1.72)	0.6 (0.26 to 1.36)	2.17 (0.13 to 35.18)
25-34 years	1.72 (1.07 to 2.78)*	1.81 (1.09 to 3.03)*	1.2 (0.68 to 2.14)	5 (0.62 to 40.37)
35-44 years	1.96 (1.23 to 3.14)**	1.57 (0.94 to 2.62)	1.18 (0.67 to 2.06)	2.17 (0.24 to 19.84)
45-54 years	3.28 (2.1 to 5.14)***	2.32 (1.43 to 3.78)***	1.85 (1.09 to 3.14)*	7.56 (0.89 to 64.15)
55-64 years	7.1 (4.62 to 10.93)***	5.02 (3.15 to 8.01)***	3.9 (2.35 to 6.48)***	8.73 (1.05 to 72.87)
65-74 years	14.87 (9.79 to 22.59)***	8.21 (5.13 to 13.14)***	6.16 (3.68 to 10.31)***	7.03 (0.82 to 60.01)
≥75 years	25.04 (16.4 to 38.23)***	13.09 (8.12 to 21.1)***	9.16 (5.41 to 15.5)***	31.77 (3.83 to 263.64)***
Sex, n (%)				
Female	1.00 (ref)	1.00 (ref)	1.00 (ref)	1.00 (ref)
Male	1.42 (1.22 to 1.67)***	1.56 (1.3 to 1.86)***	1.49 (1.22 to 1.83)***	2.12 (1.01 to 4.45)*
LFTs, cardiovascular and metabolic markers				
GGT*	1.01 (1.01 to 1.02)***	1.01 (1.01 to 1.01)***	1.01 (1.00 to 1.01)*	1.02 (0.95 to 1.08)
AST:ALT ratio	1.32 (1.26 to 1.39)***	1.17 (1.08 to 1.26)***	1.23 (1.11 to 1.36)***	0.87 (0.58 to 1.3)
Albumin	0.86 (0.85 to 0.87)***	0.9 (0.88 to 0.92)***	0.92 (0.9 to 0.94)***	0.91 (0.86 to 0.97)***
HbA1c	1.25 (1.16 to 1.35)***	1.25 (1.13 to 1.38)***	1.23 (1.07 to 1.41)***	0.89 (0.57 to 1.4)

Average systolic BP †	1.25 (1.21 to 1.29)***	1.07 (1.03 to 1.11)***	1.08 (1.03 to 1.13)***	1.21 (1.07 to 1.38)***
LDL quintiles				
First	1.00 (ref)	1.00 (ref)	1.00 (ref)	1.00 (ref)
Second	0.59 (0.46 to 0.77)***	0.67 (0.5 to 0.89)*	0.69 (0.49 to 0.96)*	0.68 (0.27 to 1.71)
Third	0.74 (0.58 to 0.95)*	0.86 (0.65 to 1.13)	0.93 (0.67 to 1.28)	0.65 (0.25 to 1.71)
Fourth	0.76 (0.6 to 0.97)*	0.89 (0.67 to 1.18)	0.93 (0.67 to 1.28)	0.77 (0.32 to 1.84)
Fifth	0.81 (0.64 to 1.01)	0.76 (0.57 to 1.01)	0.72 (0.52 to 1)	0.42 (0.13 to 1.36)
HIV status				
HIV positivity	1.41 (1.09 to 1.82)**	1.67 (1.25 to 2.22)***	1.31 (0.91 to 1.88)	1.89 (0.65 to 5.44)
HBV positivity	0.95 (0.59 to 1.52)	0.86 (0.51 to 1.45)	0.84 (0.47 to 1.51)	5.38 (2.01 to 14.42)***
BMI, n (%)				
Underweight (<18.5 kg/m^2)	1.00 (ref)	1.00 (ref)	1.00 (ref)	1.00 (ref)
Normal weight (18.5-24.9 kg/m^2)	0.63 (0.52 to 0.75)***	0.82 (0.67 to 1)	0.94 (0.75 to 1.19)	0.57 (0.3 to 1.09)
Overweight (25.0-29.9 kg/m^2)	0.56 (0.4 to 0.79)***	0.89 (0.62 to 1.29)	0.96 (0.63 to 1.47)	0.25 (0.03 to 2.01)
Obesity class (30.0 to 34.9 kg/m^2)	0.7 (0.4 to 1.23)	0.9 (0.5 to 1.63)	0.92 (0.47 to 1.82)	1.27 (0.18 to 8.93)
Obesity classes II and III (≥35 kg/m^2)	1.01 (0.44 to 2.27)	1 (0.44 to 2.28)	1.42 (0.62 to 3.3)	2.45 (0.21 to 29.07)

HR, hazards ratio; CI, confidence interval; GGT, gamma glutamyltransferase; HbA1c, hemoglobin A1c; BP, blood pressure; HIV, human immunodeficiency virus; HBV hepatitis B virus; LDL, low density lipoprotein; ALT, alanine aminotransferase; AST, aminotransferase; BMI, body mass index;.

* Hazards ratio per 10 unit increase in serum GGT.

† Hazards ratio per 10 unit increase in systolic blood pressure.

* $P < 0.05$

** $P < 0.01$

*** $P < 0.001$
